# Identifying Suicide Ideation and Suicidal Attempts in a Psychiatric Clinical Research Database using Natural Language Processing

**DOI:** 10.1038/s41598-018-25773-2

**Published:** 2018-05-09

**Authors:** Andrea C. Fernandes, Rina Dutta, Sumithra Velupillai, Jyoti Sanyal, Robert Stewart, David Chandran

**Affiliations:** 1 Institute of Psychiatry, Psychology and Neuroscience, Academic Department of Psychological Medicine, London, SE5 8AF United Kingdom; 2 UK National Institute for Health Research Biomedical Research Centre, South London and Maudsley National Health Service Foundation Trust and King’s College London, London, SE5 8AZ United Kingdom

## Abstract

Research into suicide prevention has been hampered by methodological limitations such as low sample size and recall bias. Recently, Natural Language Processing (NLP) strategies have been used with Electronic Health Records to increase information extraction from free text notes as well as structured fields concerning suicidality and this allows access to much larger cohorts than previously possible. This paper presents two novel NLP approaches – a rule-based approach to classify the presence of suicide ideation and a hybrid machine learning and rule-based approach to identify suicide attempts in a psychiatric clinical database. Good performance of the two classifiers in the evaluation study suggest they can be used to accurately detect mentions of suicide ideation and attempt within free-text documents in this psychiatric database. The novelty of the two approaches lies in the malleability of each classifier if a need to refine performance, or meet alternate classification requirements arises. The algorithms can also be adapted to fit infrastructures of other clinical datasets given sufficient clinical recording practice knowledge, without dependency on medical codes or additional data extraction of known risk factors to predict suicidal behaviour.

## Introduction

Around a million people are reported to die by suicide every year^1^ and due to the stigma associated with the nature of the death, this figure is usually assumed to be an underestimate^2^. Prevention research is generally felt to have been partially successful in achieving some reduction in suicide rates, particularly by increasing physician training and restricting access to lethal means^3^. However, recent reviews of suicide-related behaviour reported little change in prevalence estimates from the 1980s to the late 2000s^4^ and the World Health Organisation reported that around half of member countries who submitted data experienced steady or increased suicide death rates from 2000 to 2012^1,5^.

Suicide prevention research has been limited by methodological factors such as relative rarity of the event, short observation periods and recall bias^6–8^. One way to potentially overcome these limitations is through the use of large clinical datasets from which high-risk cohorts can be assessed, and the wealth of data contained in Electronic Health Records (EHRs), or clinical databases, provide a means to achieve this^4,6,9^. In addition, focusing on non-fatal suicidal behaviours, such as suicide ideation and suicide attempts, also helps to overcome some methodological limitations as their prevalence is much higher than completed suicide. Suicide ideation, is represented by the presence of current plans and wishes to attempt suicide in individuals who have not made any recent overt suicide attempts, and its severity has been proposed by Beck *et al*. as an indicator of suicidal risk^10^. Suicide attempt is defined as a non-fatal, self-directed, potentially injurious behavior with an intent to die as a result of the behavior, but that may or may not result in injury^11^. Both of these behaviours are also often considered vital risk factors for completed suicide and hence are well placed for suicide prevention research.

Previous studies using EHRs for suicide research have used structured diagnostic and International Classification of Disease, ninth revision (ICD-9) cause-of-injury codes (also known as E-codes) to identify patients who have attempted suicide^12–14^. However, the quality and practice of EHR suicidal behaviour coding varies widely. For example, a study assessing the recording of suicidal ideation and attempts in primary care clinical records (N = 15,761) found 1,025 patients who had suicidal ideation recorded in text fields, of whom only 3% had a structured code indicative of this. Furthermore of 86 patients identified as having made a suicide attempt, only 19% had the relevant code for this^15^.

Natural Language Processing (NLP) offers wide-ranging solutions to retrieve and classify data from EHRs. Text mining, which is part of the NLP family, is defined as the analysis of ‘naturally-occurring’ text driven by human specification to achieve one or a range of goals (e.g. information retrieval or artificial intelligence)^16^. The analysis often manifests as a set of programming rules or machine learning algorithms (i.e. algorithms generated from automated learning from manual examples), whose eventual output should represent human output as much as possible^17^. One of its applications can be to identify and classify instances of suicide ideation, attempts and death by suicide recorded within free-text medical notes, if the data are not readily derived from structured fields^18^. This use alone can significantly improve the common limitation in suicide prevention research of low case sample sizes.

Using NLP (and text mining) is a relatively new venture in suicide prevention research^14,19,20^ and only recently have some studies reported using intuitive text mining approaches to identify suicidal behaviour in clinical notes^21–23^. Text mining methods used in suicide research have evolved from simplistic dictionaries and search engines^18,24,25^ to more NLP orientated approaches. Haerian *et al*., described a hybrid process^26^ developed for radiology databases by Friedman *et al*.^27,28^, combining use of structured E-codes and NLP to identify suicide thoughts and attempts in their clinical EHR database. In 2014, Ben-Ari *et al*. integrated their simple text search approach with a random forest classifier to identify suicide attempts in clinical notes^23^. More recently, Metzger *et al*. utilised seven standard machine learning techniques (Support Vector Machines, predictive association rules, decision trees, logistic regression, Naïve Bayes, random forest and neural networks) to classify suicidal ideation from suicide attempt from a list of demographic and clinical variables^22^.

Contributing to the sparse literature on text mining methods used to identify suicidal behaviour for research^4,29^, the aim of this study is to describe the independent development of two NLP tools – one for detecting the presence of recorded suicide ideation and the other for detecting a recorded suicide attempt – and to evaluate each tool’s performance against manual text annotation using precision and recall. To our knowledge, this is the first time rules-based or machine learning have been used to detect suicidality in a psychiatric database. We seek to present the strengths and weaknesses of our approaches, with a view to future development and improvement of text analysis in this topic area. We recognize that there are no standard rules outlining how to use text mining techniques to extract data from observational datasets and so, in this manuscript, we present two working approaches.

## Results

**Figure 1:**
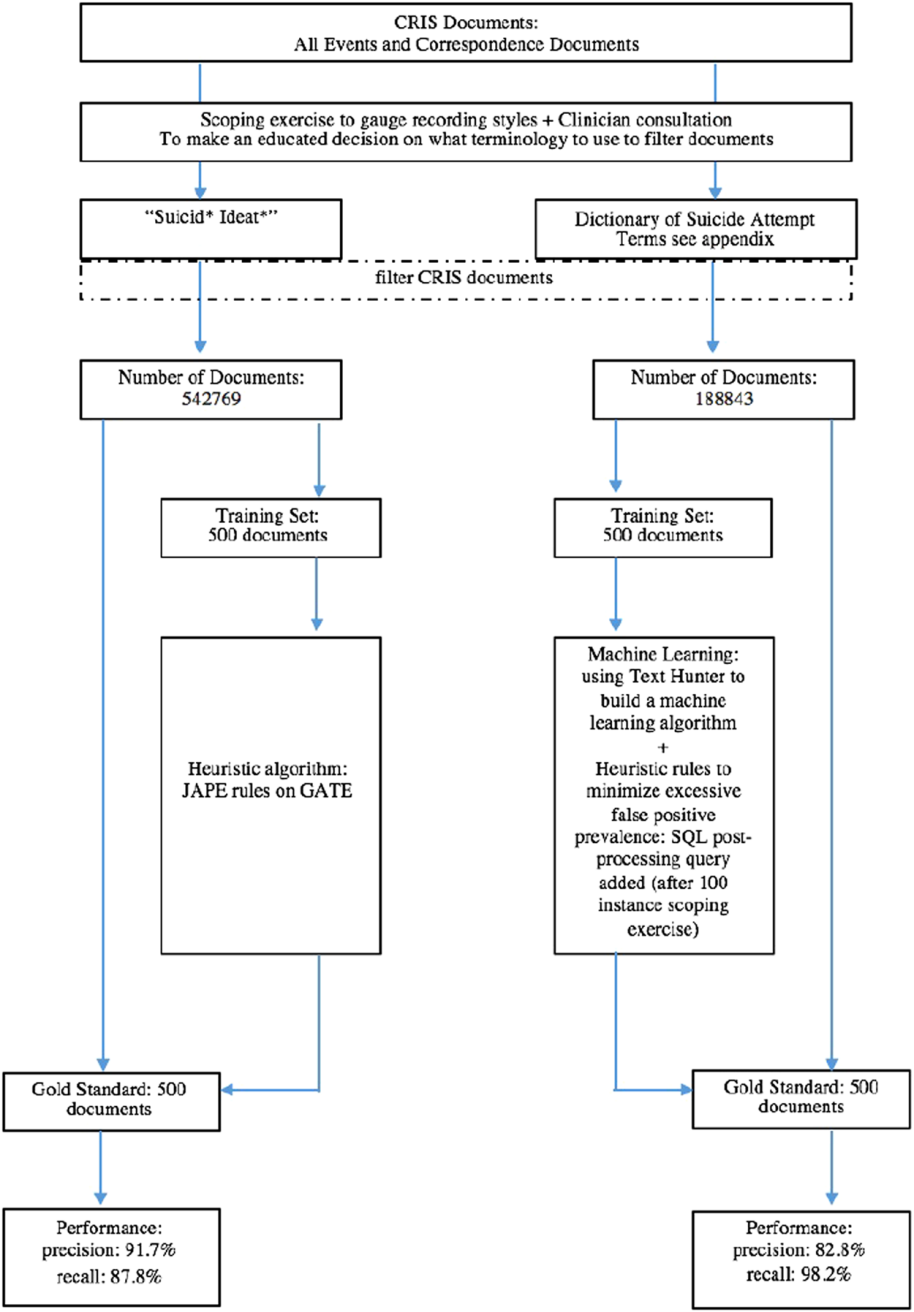
Schematic Outline of classification tool development and evaluation study. Event notes are free-text fields, where day-to-day notes can be recorded in any layout or format. Correspondence notes are used to attach any formal correspondences between patient and clinical staff or between clinical staff members. These documents constitute of letters and questionnaires recorded in word documents (which included any electronic questionnaires administered to the patient), pdf documents and any other documents related to the patient. “Suicid* ideat*” is the text pattern used to filter variations of the phrase “suicidal ideation”, within each sentence in the clinical notes, where the asterisk denotes a wild card allowing for any combination of letters in the word after the initial specified sequence.

Two text mining applications were developed and evaluated the results of which is described in detail below. A schematic flow diagram of the of the development process and evaluation process is provided in Fig. 1 and further details on the description of data used and study designs are provided in Section 4: Methods.

### Document Characteristics

Of the 500 documents to generate the gold standard set for suicide ideation, there were 459 instances from events, 41 from correspondence notes. Similarly, of the 500 documents to generate the gold standard for suicide attempt there were 275 number of instances from events, 225 from correspondence notes. There were 9 documents with mentions of self-harm (“attempts at self-harm”, “Patient reports that the overdose was not a suicide attempt but a way to ‘stop’ the stress”, “because at the time she had been expressing thoughts that life was not worth living, it [the self-harm incident] was interpreted as an attempt to end her life”, “has claimed cutting himself to achieve relief from stress. …denied that this was an attempt to kill himself”, “He reported to us that they were done with the desire to harm himself but weren’t suicide attempts.”, “suicide attempts without clear expectation of death”, “Patient states that she ‘wants to die’ but says that her overdose was not a suicide attempt”, “Has taken 4 tablets of Paracetamol in the past when feeling sad but not in an attempt to end her life” and “but [patient] able to say it [self-harm incident] was not a suicide attempt”).

During the training stage of the development of the suicide attempt classification tool a disproportionately high number of subject titles and neutral mentions of suicide attempt was evident (e.g. “*Suicide attempts/ideation*” or “b) Suicide attempts x” or “History of previous suicide attempts*”), which would bias the of the algorithm preventing a fair evaluation. To demonstrate the bias, the author evaluated 100 annotations and found precision of 66.7% and a recall of 98.3% indicating good identification of actual suicide attempts but a high number of incorrectly identified suicide attempts (i.e. neutral mentions of suicide attempt being identified as an actual suicide attempt). As a resolution, a post-processing rule-based SQL filter was applied to the machine learning results to exclude neutral mentions of suicide attempt – See GitHub link for post-processing SQL filter (GitHub link: https://github.com/andreafernandes/NLP_Tools_Development). Hereafter the machine learning algorithm will be called the hybrid machine learning and rule-based algorithm (the ‘hybrid algorithm’).

### Performance of each NLP Classification tool: Precision and Recall

The performance was evaluated for each NLP tool separately as the different approaches prevented comparison between the NLP tools. Each NLP classification tool was evaluated against a manually annotated gold standard set producing precision and recall statistics.

Of a total of 500 instances with the mention “suicid* ideat*” with 302 instances indicating patient had experienced suicide ideation, the rules based algorithm correctly identified 265 and incorrectly identified 24 instances giving a sensitivity of 87.8% and a precision of 91.7% (Table 1). Of a total of 500 instances mentioning any of the dictionary terms of suicide attempt (See Supplementary Table S2 in GitHub link: https://github.com/andreafernandes/NLP_Tools_Development for details) with 388 instances indicating patient had attempted suicide, the ‘hybrid algorithm’ correctly identified 381 and incorrectly identified 79 instances giving a sensitivity of 98.2% and a precision of 82.8% (Table 2).

**Table 1:** Classifier Performance Results for Suicide Ideation (n = 500 instances).

Classifier	Gold Standard	
	True Event	Non-True Event
True Event	a = 265	c = 24
Non-True Event	b = 37	d = 174
**Performance Results**		
Precision (PPV) (a/a + c)	91.7%	
Recall (Sensitivity) (a/a + b)	87.8%

**Table 2:** Classifier Performance Results for Suicide Attempt after Post-Processing (n = 500).

Classifier	Gold Standard	
	Suicide Attempt	Not Suicide Attempt
Suicide Attempt	a = 381	c = 79
Not Suicide Attempt	b = 7	d = 33
**Performance Results**		
Precision (PPV) (a/a + c)	82.8%	
Recall (Sensitivity) (a/a + b)	98.2%	

## Discussion

We have developed two text mining applications designed to extract quantitative data from psychiatric clinical text. Two distinct NLP approaches are described to identify and classify suicide ideation and attempts, both of which performed well as indicated by high precision and recall statistics. This is one of few current studies exploring and applying text mining software to specifically build classification algorithms within a large psychiatric database to detect suicidal ideation and attempts^29,30^.

Although previous research is sparse, the performance of our NLP tools resonates with other published studies using bespoke NLP techniques and machine learning NLP tools to classify suicide–related ideation and attempt data. We found a precision of 91.7% for identifying suicide ideation and 82.8% for identifying suicide attempts. A study comparing the use of structured ICD-9 (E950–959) codes versus using suicidality specific concept unique identifiers generated from an NLP process to identify patients with suicidal thoughts or behaviours, found a precision of 97% (50 patient records used in training, 280 patient records were used as gold standard) using ICD-9 codes together with NLP approach, versus 60% using just the NLP algorithm^26^. Another study used accident and emergency records to extract data on patient demographics, clinical observation and laboratory test data to classify (or predict) suicide attempts using seven machine learning techniques (namely, predictive association rules, decision trees, neural networks, logistic regression, random forest, naïve Bayes and support vector machine). They found random forest and naïve Bayes (two examples of machine learning classification methods used in NLP) optimally represented results from manually calculated suicide attempt prevalence, with precision of 93.1% and 96.9% respectively^22^ (483 patient records, with 112 suicide attempts in the training set and 438 patient records in the test set).

It is difficult to compare these two studies with our study due to major methodological differences, however both studies represent innovative means of detecting suicidal and self-harm behaviour. The relatively high precision values reported in each paper could be owing to the algorithms being designed around a small and precise set of cases, which may not be generalizable to larger datasets. Metzger *et al*. reported use of low numbers of cases in training sets while Haerian *et al*. did not report how many cases were present within the training set of 50 patients. Both studies also reported difficulty in contextualising the identified suicidality concept and hence ruling out false positive mentions (e.g. distinguishing instances of self-harm from suicide attempts). In addition, the dependence on using structured codes together with NLP algorithm means the text mining process may perform poorly within datasets that do not use structured ICD codes to record suicidality (as shown in their results). Metzger *et al*. conducted the study in an emergency site without any available psychiatric services which may introduce bias in the predictive model refraining it from being applicable to other datasets. Moreover, the reliance on several patient clinical features in this model weakens the usability of the algorithm in the absence of such data. However, Metzger *et al*. and Haerian *et al*. provide a robust means with which to extract or identify suicidal behaviour from observational datasets. We argue that our tools offer further malleability and adaptability to other databases and add to the intuition of the methods presented by Metzger *et al*. and Haerian *et al*.

There are limitations to our approaches. Firstly, in the definition of each suicidality concept (i.e. basic definition of ideation and a comprehensive definition of suicide attempts) and secondly, in the choice of the NLP techniques (i.e. rule-based for suicide ideation, and hybrid machine learning and rule-based for suicide attempt). For the suicidal ideation classifier, the use of basic rules (i.e. sentences containing the terms “suicid*” and “ideat*”) to identify suicide ideation restricts the model from detecting other permutations and variations in text describing suicide ideation. While it is acknowledged that the JAPE code written to identify sentences with “suicid*” and “ideat*” mentions and classify them could be deemed too simplistic, using a lengthy list of terms, as we did to identify suicide attempt, runs the risk of increasing false positive instances making it harder to train models as we see with the requirement for further refinement to optimise the suicide attempt classifier. This refinement will vary with databases making our algorithm less generalizable to other databases not accompanied with text mining expertise. On the other hand, using a single permutation such as “suicid*” followed by “ideat*” is justified because the term “suicide/al ideation” is a standard term used in clinical and research settings and hence is recorded in clinical notes along with “suicidal thoughts” making the latter redundant. It is noteworthy to mention the attempt to extract suicide attempt data separate from self-harm. While there are differences in the definition of a suicide attempt and self-harm, in reality when recording these differences are not as distinct. Another limitation is the sole use of SVM as a classifier model within a pre-defined framework. Some studies argue that such models are too complex to be understood by untrained researchers and the underlying classification mechanism is not sufficiently clear to easily allow for edits^31^. Moreover, other machine learning algorithms, such as random forests or Naïve Bayes, have been applied successfully in similar tasks, and could be worth investigating further. Finally, a limitation of both classifiers is the use of non-standardised dictionaries. The dictionary to define a suicide attempt was generated after a thorough analysis of the patient records and clinical advice. These dictionaries could limit the generalisability of the algorithms to other databases.

We recognize that further training iterations with TextHunter with larger corpora for the classifier might have further improved the performance of the tool. However due to the excessive numbers of neutral mentions of suicide attempt instances (for example, in the form of questions in questionnaire, e.g. “Have you ever attempted suicide?” or subheadings, e.g. “Past suicide attempts”), we chose instead to develop the post-processing rules (i.e. to exclude the excessive generic neutral mentions of suicide attempt). We also appreciate that the heuristics could be applied prior to training and testing set generation. Moreover, instead of adding a post-processing step with heuristics to mitigate classifier errors, an alternative would be to design the classification task with an appropriate sample for the machine learning algorithm to learn from.

Though these NLP tools enable us to harness the potential of using large longitudinal datasets to explore suicidal behaviour from new perspectives, during their development, there is always a decision to be made on how we define our variable of interest (in this case, symptom or behaviour) and then what the best NLP approach is to ensure we are able to extract the appropriate data with good sensitivity and specificity. Do we identify all variations of a given variable (quantity) or do we want to identify the most common occurrence of the variable (quality)? We opted for the latter to develop a suicide ideation NLP tool (to take into account the fairly standard terminology used by clinicians to record presence or absence of suicide ideation) and the former for suicide attempts (to encapsulate the various ways in which suicide attempt is mentioned in the psychiatric clinical notes in this database). We postulate that using a bespoke dictionary makes the classification tool more adaptable to the richness of the dataset and recording practices. Given the performance for both classifiers, they fulfil the objective for which they were designed which was to identify patients who have ever experienced suicidal ideation or suicide attempt.

There are clear strengths to both the text mining approaches reported here. These NLP tools were built to suit this particular observation dataset and with the primary aim to maximise data capture on suicidal behaviour written in free-text notes with good precision and recall. The results from our evaluation study demonstrate good performance and larger case sample detection. The NLP tools developed in our approach do not rely on any codes or structured fields to function, which broadens the scope for retrieving data from other textual fields. Though we would not propose that our tool would be applied without modification to another clinical setting, for any external applicability, the infrastructure of the classification models allows for development and, more importantly, adaptability to different datasets and researcher objectives given sufficient clinical knowledge of recording styles of suicidal behaviour. For example, the dictionaries can be easily updated to include typos or common clinical recording phrases. In addition, this study provides some insight in using an intricate and simplistic approach to detect suicidality and depending on the complexity of data in other databases either methodology can be used to ensure optimal detection. In addition, the algorithm independently detects mentions of suicidal ideation or attempts as opposed to using further variables to predict such behaviour (i.e. predictive modelling), which may require further text mining.

The risk of suicide is many times higher among patients with a mental health diagnosis compared to the general population^32^. Hence our training set is likely to have contained a sufficient range of examples of how suicidal ideation or attempts are mentioned, as well as ample cohort sizes for testing models. The large database and corpus of suicide attempt instances allowed us to distinguish between self-harm and suicide attempt and exclude the former when developing classification models. The decision to exclude self-harm was purely based on clinical differences between suicide attempt and self-harm behaviour, where the former is done with intention to die (regardless of lethality) and the latter may manifest as an act or cry for help/attention or there can be many other reasons why one self-harms. There may be different risk factors or motivations for each behaviour to manifest and different treatments are choses to resolve each of the disorders. Hence it is important to have a distinction between the two behaviours. On assessment of the proportion of self-harm instances detected as false positives, we found nine instances out of 500. Finally, GATE, provides a user-friendly platform to refine the performance of the algorithm to achieve optimal precision and recall, allowing improvement on weaknesses in the algorithm, dictionaries or definition of the data variable.

**Figure 2:**
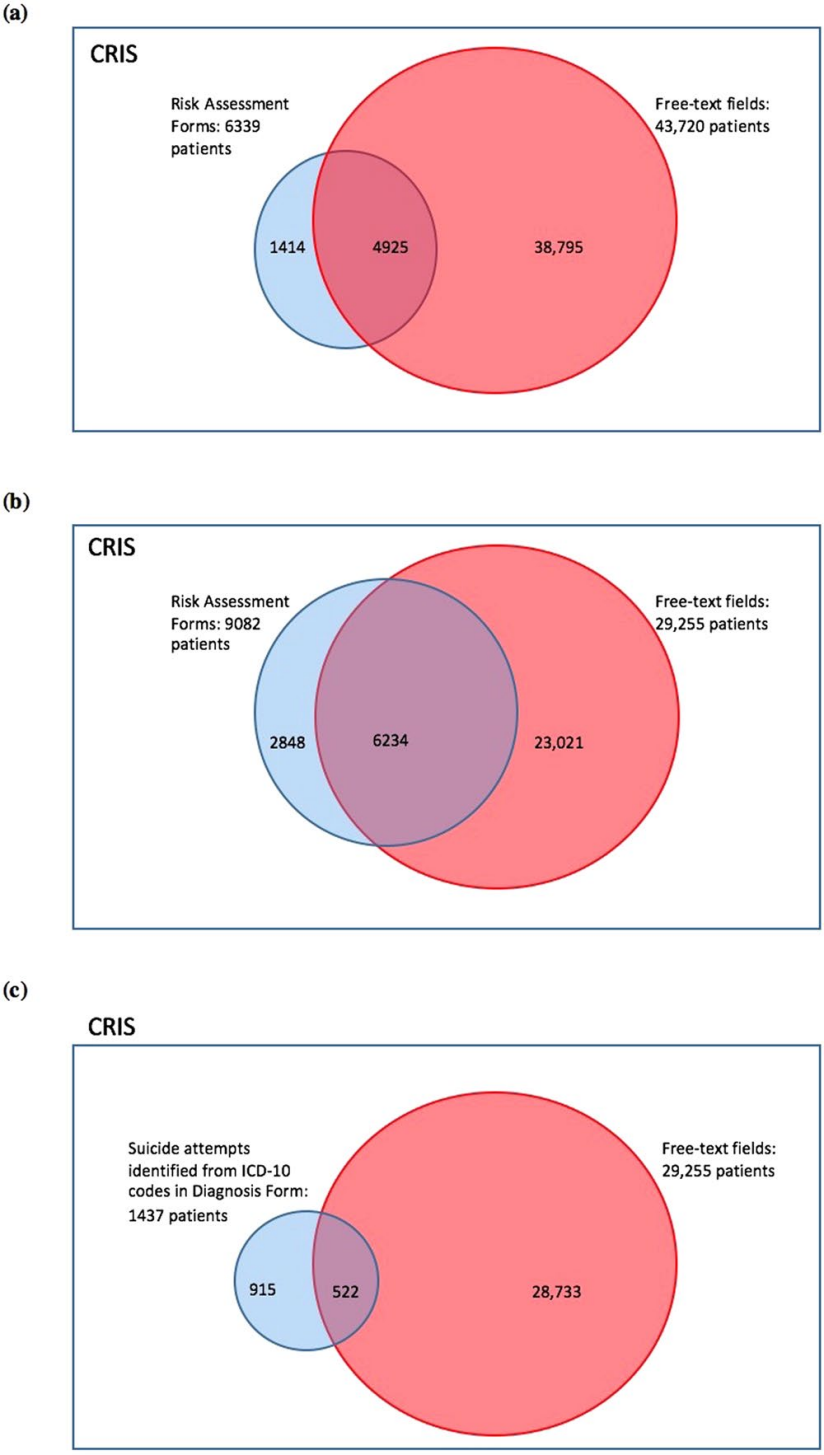
Venn diagrams comparing patient numbers obtained when (**a**) using NLP to identify suicide ideation versus using Risk Assessment (structured) fields only; (**b**) using NLP to identify suicide attempts versus using Risk Assessment (structured) fields only and (**c**) using NLP to identify suicide attempt versus using ICD-10 codes for suicide attempt only.

Of relevance to the NLP models developed in this project, there are existing structured fields within the patient EHR that are dedicated to the recording of suicidal behaviours. In-depth risk assessment schedules, which themselves include binary ‘tick box’ fields, indicating the presence or not of previous/current suicide ideation and suicide attempt and structured ICD-10 diagnostic fields are designed to capture patient suicidal ideation experiences or attempts of suicide. However their relatively lower usage compared to Event and Correspondence note fields, give impetus to design NLP tools to detect suicidal ideation or attempts recorded elsewhere. In a small study conducted to demonstrate this lower usage we compared the number of patients identified via use of structured fields only with the number of patients identified via use of our NLP tools. Figure 2 shows diagrammatically the increased number of patients identified when using the NLP tools to identify patients versus when using dedicated structured fields alone. Previously, Neuman *et al*. have described the use of clinical text mining as a proactive approach, overcoming limits of selective use of questionnaires or structured forms which need to be administered to patients to screen for depression^33^. They described a text mining software that detects the mention of depression in web-texts and further uses lexical analysis to increase precision in detection of depressive episodes. The algorithm can be used as a screening to alert web-users to seek further advice. Sohn *et al*. described the development of a hybrid rule-based and machine learning approach to identify as many adverse drug reactions from clinical notes as possible so as not to rely on manual chart reviews^34^, which are “time-consuming and effort-intensive” for routine use. In addition, Castro *et al*. highlight the importance of using NLP techniques within EHRs to avoid labour intensive and costly methods to validate potentially misclassified diagnoses recorded in structured forms. In this regard, our results also support use of text mining extracted data over use of structured form extracted data^35^. While one approach to improve data availability might be to constrain clinicians to complete structured fields in EHRs, this process can promote automaticity and shift the focus away from deep probing and analytical clinical thinking^36^. We believe that NLP will continue to grow in value, as from both clinical communication and medicolegal perspectives, narrative text will remain integral to psychiatric EHRs, particularly for descriptions of each patient’s symptoms and their mental state.

Similar to our study, Castro *et al*. apply a machine learning approach and a rule-based approach to identifying bipolar disorder^35^. They also concluded that either method was as good as manual identification of bipolar disorder. Unlike our findings, the rule-based approach required use of a more relaxed set of “non-hierarchical” rules to improve performance. This study adds to the rationale that NLP tools may require some processing and modification to work to their optimal performance. Bearing in mind future directions, in terms of development of the reported classifiers, there is scope for further work to (1) robustly modify the tools to withstand being developed as rules and hybrid machine learning approach and provide optimal evaluation and (2) to develop temporal reasoning solutions in order to distinguish suicidal behaviour at different time points, which will be beneficial for longitudinal observation and analysis. The current machine learning and rule-based hybrid classification algorithm to detect suicide attempts could benefit from the incorporation of lexical and semantic analysis where further machine-learning, dictionaries and exclusion rules can be implemented to improve word-to-concept relations^33^. Both the classification algorithms should have applicability, with or without further adaptation, to databases utilised for research on emotion detection within suicide notes^24,37^ and for predicting suicidal behaviour using retrospective risk factor data from EHRs^7^. Previously, both areas of research have incorporated NLP techniques on occasions but have had limitations including small sample sizes. Moreover, there are calls to use mixed methodology to make progress in suicide research, integrating hypotheses generated from qualitative analyses with quantitative exploration,^38^ using text from large EHR cohorts together with NLP data identifying suicide attempts; such research is potentially now more feasible. Finally, such NLP classification software might support clinical decision support algorithms, combining patient-reported outcomes, health-related information and clinical observations to assist clinicians in providing optimal suicide prevention care options^20^. There are potential applications in routine clinical surveillance and service evaluation. For example, Neuman *et al*. describe one utility of their text mining algorithm (which screens for depression within web-texts) as a flag system for individuals who may have a significant depressive disorder and need referral to psychiatric care services^33^.

We have described two NLP solutions to identify suicide ideation and suicide attempt in a psychiatric database, both of which performed well as classifiers. Each of our classifiers enables progress of research which would otherwise be limited to low sample size and lack of statistical power. In our case, the data extracted from each classifier will be used to contribute to research investigating the association of antidepressant use with suicide ideation and suicide attempt. There are clear challenges with using and developing NLP processes to identify suicidality within clinical notes - from recording practices and defining suicidality concepts, to building classification algorithms, evaluation and refinement. The field of using NLP to identify suicidality is still in its infancy and so sharing our methods can be used towards advancements in this field to further progress suicide prevention research.

## Methods

### The Data Source

The Clinical Record Interactive Search (CRIS) system provides de-identified information sourced from South London and Maudsley (SLaM) NHS Trust, a secondary and tertiary mental healthcare provider serving a geographic catchment of roughly 1.3 million residents of four south London boroughs (Lambeth, Southwark, Lewisham, and Croydon). EHRs have been used comprehensively across all SLaM services since 2006. CRIS was established in 2008 to allow searching and retrieval of de-identified clinical information for research purposes^39,40^ within a robust, patient-led governance framework^41^ and currently houses records on ~250,000 patients receiving care. The system allows for retrieval of information from de-identified free-text fields, as well as structured fields, and the use of CRIS for research was approved by the Oxfordshire Research Ethics Committee C (reference 08/H0606/71 + 5).

The documents used in this study are Events and Correspondence. These two document types constitute more than 60% of all patient related notes, and are recorded by clinical staff and community carers. Hence they pose as optimal sources of data to generate machine learning or rule-based algorithms. Event notes are free-text fields, where day-to-day notes can be recorded in any layout or format. Correspondence notes are used to attach any formal correspondences between patient and clinical staff or between clinical staff members. These documents constitute of letters and questionnaires recorded in word documents (which included any electronic questionnaires administered to the patient), pdf documents and any other documents related to the patient.

### The Study Design

Figure 1 shows a schematic representation of the study design for developing and evaluating each NLP tool.

As there are over a million events and correspondence documents within the CRIS database, all relating to patient symptoms and experiences, in order to create NLP tools to identify suicide attempt and suicide ideation it was important to filter the documents to leave only those which contained some mention of these phenomena: otherwise the documents required for training would be excessively large. For training each algorithm, a set of 500 documents constituting of events and correspondence notes were selected from a total of 542,769 documents from the suicide attempt cohort and 188,843 documents from the suicide ideation cohort (Fig. 1). The decision to select 500 documents as sufficient was made after several informal scoping exercises and after discussing with the on-site NLP expert with experience using the CRIS dataset to develop previous text mining algorithms. The sparse literature suggests no standard rules for determining sizes of gold standard and training sets. One method of determining the size of gold standard/training corpus is by Juckett *et al*., 2012; however, that paper also mentions how most studies decide on a gold standard or training size purely by ad hoc reasoning depending on the data, financial, time or personnel constraints^42^.

### Natural Language Processing Software

The development of the classification models for suicide ideation and attempt was conducted using NLP software (Fig. 1) on the General Architecture for Text Engineering platform (GATE; www.gate.ac.uk) which allows researchers to pre-process, classify and evaluate classification models. GATE pre-processing involves adding features to selected corpora such as tokenisers, sentence splitters, part-of-speech taggers and semantic taggers to help with classification. Classification models can then be generated via two means: using rule-based pattern matching programming languages (two major ones being a java-based language specific to GATE, called JAPE, and Groovy) or using machine learning algorithms, provided within the Batch Learning plugin supplied with GATE^43^. This plugin consists of different machine learning classification tools such as regression analysis, random forest, k-means clustering and Support Vector Machines (SVMs). Evaluation can be conducted using precision, recall and F1 measures, in addition to graphic comparisons of annotation sets between annotators.

A user-friendly in-house software called TextHunter^44^ was additionally developed as a single platform to host the machine learning component provided by GATE. It provides a full end-to-end process incorporating document retrieval, sentence-level processing, extracting relevant instances within clinical text, creating training data, hosting and running the machine learning element (training), creating the gold standard set, configuring and testing the machine learning algorithm and further active learning for refinement of the model.

TextHunter builds and evaluates a range of models against the task, using different features and SVM parameters each time. The default feature vector used by TextHunter is a classic bag of words using part-of-speech tags and token stems from the user specified context around a concept. When applying a model to unseen data, TextHunter creates feature vectors from up to six different combinations of sentences around the sentence containing the concept term. The classification resulting from the feature vector producing the highest overall confidence is chosen as the result. TextHunter also takes features of the GATE implementation of the ConText algorithm^45^, which uses hand crafted rules to determine whether a concept is negated, temporally irrelevant or refers to a subject other than the patient. Stop word removal is also explored during feature selection. Cross validation of the training data is used to mitigate the dangers of overfitting the model to a small amount of data. The model producing the best F1 score is taken forward for testing against the human labeled ‘test’ corpus, which is never used in model training. In addition, for each record, TextHunter returns a probability that it was classified correctly. Therefore, using the records with the lowest probabilities, developers can supplement the gold standard, identifying where the algorithm has correctly or incorrectly classified records. Through this further training can be done and then the algorithm can be re-evaluated and re-run. This software was used to build a NLP tool for suicide attempt.

### Building a Rule-based classification model for Suicide Ideation

Suicide ideation was classified using JAPE coding and a set of dictionaries within GATE (i.e. a rule-based approach). The JAPE code was written to reflect the main aspects of the rules generated to classify suicide ideation. A total of 500 documents in the training set were used to build and test the JAPE rules. The code was continually tested, reviewed and refined until the code suitably reflected the classification rules and objectives. It describes the text pattern used to filter variations of the phrase “suicidal ideation”, within each sentence in the clinical notes, where the asterisk denotes a wild card allowing for any combination of letters in the word after the initial specified sequence.

Two negation dictionaries (gazetteers) were created, which if mentioned in the sentence would negate the mentioned suicidal ideation. The use of the term “suicid*” and “ideat*” in the same sentence was sufficient to fulfil our primary goal of identifying patients who had ever had suicidal ideation recorded in their notes. (GitHub link: https://github.com/andreafernandes/NLP_Tools_Development).

### Building a machine learning classification model for Suicide Attempt

For suicide attempt, a list of terms was used to identify and extract documents potentially mentioning instances of suicide attempts. The list of terms defining suicide attempt was gathered manually using three sources: i) suicide epidemiology literature^12,26^, ii) a random selection of 150 documents from patients who were recorded as having had a past suicide attempt in risk assessment forms, were screened for mentions of suicide attempt to capture clinical habits of recording this, and iii) clinician suggestions (JD, JLM and DO). Further refinement of the list was conducted upon first test-run of the classifier algorithm (see supplementary material for final list of terms used to define suicide attempt: https://github.com/andreafernandes/NLP_Tools_Development).

Using the TextHunter application all documents with the specified terms were identified and, using the application’s in-built SVM classifier, a model was trained to identify a true suicide attempt from irrelevant or negation statements depending on the context of the sentence. A training set of 500 annotations was used to develop the model. During training of the machine learning tool, we noticed that there were many instances of neutral mentions of suicide attempt which may influence eventual performance of the algorithm. To assess this, a scoping exercise of 100 instances was conducted to demonstrate how these instances would have affected final performance. The results of the scoping exercise show that the suicide attempt machine learning NLP tool identified true suicide attempts well but could not distinguish non-true suicide attempt events from true events and over-classified them as true events which is reflected in the low precision value (Precision – 66.7%; Recall – 98.3%). In order to improve on this performance, the suicide attempt classifier was re-evaluated after the following post-processing heuristics were applied to the tool.

#### Post-processing of the machine learning Suicide Attempt classification model

The hybrid machine learning suicide and rule-based attempt NLP tool generated several fields as output, two of them being document *contextString* (this field brings back an excerpt – about 500 characters long - which contains the phrase which the classifier deemed to mean a true or non-true suicide attempt) and *match* (this field denotes what text was matched that the classifier deemed true or non-true suicide attempt). Using *contextString* and *match*, a post-processing SQL query was generated to improve the classifier’s performance. For post-processing, we examined a random selection of 500 annotations of the suicide attempt classifier and identified text patterns, phrases or words of the incorrectly annotated instances (See GitHub link for SQL code: https://github.com/andreafernandes/NLP_Tools_Development). These instances were then excluded from the model and performance was re-measured against the gold standard. Post-processing of the suicide attempt classification model involved exclusion of irrelevant phrases such as title phrases from generic questionnaires, redundant ambiguous phrases such as “previously also attempted to” and phrases containing negation preceding the actual suicide attempt term match (e.g. ‘ZZZZZ said she would **not** [negation] *attempt to take her life* [suicide attempt term]’). The performance results improved to a higher level (Table 2) after this post-processing step.

### Inter-rater agreement, Generating Gold Standard and Training datasets

Based on an agreed set of classification rules, the generation of gold standard sets for both Ideation and Attempts were conducted by RD, RS and AF (RD and RS in their capacity as senior psychiatrists who actively utilise and record patient notes onto the clinical system). Notes supplied in the supplementary material summarises rules that were followed to create gold standard sets for suicidal ideation and attempt. The sets were independently classified by all three authors with good inter-rater agreement as indicated by a Cohen’s kappa of 0.85 for suicide ideation and 0.86 for suicide attempt (See supplementary material for further details on inter-rater agreement and rules: https://github.com/andreafernandes/NLP_Tools_Development). The gold standard sets for suicidal ideation and attempts were then annotated by AF and RD, to test the classification algorithms developed. A total of 500 documents each were annotated for each gold standard set.

### Testing the Algorithms

The model’s performance was tested by measuring the results from the model against the results from manual annotations (the gold standard dataset) providing precision (positive predictive value) and recall (sensitivity) statistics. Precision was defined as the proportion of correctly identified true events over the total number of true events identified by the classifier. Recall was defined as the proportion of true events identified by the classifier identified over the total number of actual true events (identified by manual annotation). For suicide attempt, the evaluation was conducted after the application of the post-processing heuristics.

### Availability of Materials and Data

The datasets generated during and/or analysed during the current study are based on pseudonymised patient data which is not publicly available. Access to this data requires a formal application procedure to the patient data oversight committee. On reasonable request and after suitable arrangements, data and the text mining procedures employed here can be viewed within the secure dataset firewall.
